# Evaluation and treatment of compression neuropathies of the upper limb

**DOI:** 10.1186/1753-6561-9-S3-A110

**Published:** 2015-05-19

**Authors:** Sarah Ewald

**Affiliations:** 1 City Handtherapie, Zurich, 8006, Switzerland

## 

Compression neuropathies of the upper extremity can manifest with an obvious set of symptoms that are easily identified, such as with carpal tunnel syndrome (CTS). They can also appear with diffuse symptoms so similar or in coexistence with other conditions that they can be a challenge to identify, as is often the case in radial tunnel syndrome which closely resembles lateral epicondylitis in presentation [1]. Compression neuropathies are a dynamic mechanical phenomenon occurring when a nerve is compressed under a specific anatomical structure such as an opening in a fibrous or muscular tissue or a fibro-osseous tunnel [2].

The symptoms a patient experiences can be diffuse and variable, and may include muscle weakness, sensory paresthesia, pain, aching and fatigue in the extremity. Initially, a change in position may relieve the compression or alter the area of the nerve that is compressed, in which case the symptoms subside or change. As the compression persists the symptoms become more lasting and can no longer be relieved by an alteration in position. In response to the compressionthe nerve begins to change, progressing from a breakdown of the blood nerve barrier, connective tissue thickening, demyelination and ultimately to axonal degeneration [3]. Untreated, compression neuropathies, can result in permanent sensory loss and /or muscle atrophy, both ofwhich significantly impact function.

Conservative treatment of compression neuropathies is multifaceted and may include activity analysis, patient education, ergonomic evaluation, postural exercises, stretching of muscles, nerve gliding exercises, splinting, taping, use of modalities such as ultrasound or iontophoresis [4], strengthening and improvement of aerobic capacity. Conservative treatment requires a careful, balanced, step by step approach on the part of the therapist and the patient. ”Aggressive stretching, particularly of neural tissue will needlessly exacerbate symptoms.”([3] page 633). Patient education is essential for this process to be effective.

Success rates for conservative interventions vary. Shah et al. [5] reported an 88% success rate when treating patients with cubital tunnel using splinting and activity modification. Povlsen et al. [6] reported upon long-term results of CTS patients treated with wrist splinting, patient satisfaction was high and a 43% resolution rate was reported. In a group of 197 patients, Rosmaryn [7] compared splinting for CTS to splinting and an exercise program with tendon and nerve gliding exercises and found that the splinting and exercise program was more effective than splinting alone in the conservative treatment of CTS.

This workshop will present an overview of upper extremity entrapment neuropathies: the anatomical structures that are involved, the clinical picture of compression neuropathies of the radial, median and ulnar nerves, assessment and therapeutic treatment methods.
